# Trophic Chain Organochlorine Pesticide Contamination in a Highly Productive Upwelling Area in Southeastern Brazil

**DOI:** 10.3390/ijerph20146343

**Published:** 2023-07-11

**Authors:** Ricardo Cavalcanti Lavandier, Jennifer Arêas, Leila Soledade Lemos, Jailson Fulgêncio de Moura, Satie Taniguchi, Rosalinda Montone, Natalia Soares Quinete, Rachel Ann Hauser-Davis, Salvatore Siciliano, Isabel Moreira

**Affiliations:** 1 Departamento de Química, Pontifícia Universidade Católica do Rio de Janeiro (PUC-Rio), Rua Marquês de São Vicente, 225, Gávea, Rio de Janeiro 22453-900, Brazil; 2 PIBIC/Fundação Oswaldo Cruz, Av. Brasil, 4.365, Manguinhos, Rio de Janeiro 21040-900, Brazil; 3 Institute of Environment, Florida International University, North Miami, FL 33181, USA; 4 Department of Chemistry and Biochemistry, Florida International University, Miami, FL 33199, USA; 5 Systems Ecology, Leibniz Center for Tropical Marine Ecology (ZMT), Fahrenheitstrasse 6, 28359 Bremen, Germany; 6 Instituto Oceanográfico, Universidade de São Paulo (USP), Praça do Oceanográfico, 191, Butantã, São Paulo 05508-120, Brazil; 7 Laboratório de Avaliação e Promoção da Saúde Ambiental, Instituto Oswaldo Cruz/Fundação Oswaldo Cruz, Av. Brasil, 4.365, Manguinhos, Rio de Janeiro 21040-900, Brazil; 8 Departamento de Ciências Biológicas, Escola Nacional de Saúde Pública/Fundação Oswaldo Cruz, Rua Leopoldo Bulhões, 1.480, Manguinhos, Rio de Janeiro 20911-300, Brazil

**Keywords:** OCP, biomagnification, fish, cetacean, *Pontoporia blainvillei*, Rio de Janeiro, DDT metabolites

## Abstract

Organochlorine pesticides (OCP) are legacy anthropogenic compounds known to persist for several years in the environment. The continuous use of some OCP, such as DDT, after restrictions in developing countries are cause of concern, due to their deleterious effects to marine life and humans. Studies assessing OCP contamination in coastal environments are still scarce in South America and there is a need to understand the impacts from trophic chain accumulation of these pollutants in marine life. In this study, we have assessed OCP levels in muscle and liver and estimated the biomagnification factor in several upwelling system trophic chain members, including fish, squid, and marine mammal from Southeastern Brazil. DDT degradation product DDE was the OCP detected in the highest concentrations in Franciscana dolphins (*Pontoporia blainvillei*), 86.4 ng·g^−1^ wet weight, and fish muscle and liver. In general, higher OCP levels were found in liver than in muscle, except for croaker. Biomagnification factors (BMF) of OCP in the top predator *P. blainvillei* and the carnivorous cutlass fish (*Trichiurus lepturus*) were on average between 0.2 and 1.8. Continued OCP monitoring in this region is warranted to better understand the distribution and fate of these compounds over time, with the goal to establish strategies for the conservation of local dolphin species and to assess human health risks from local coastal region populations.

## 1. Introduction

Organochlorine pesticides (OCP) are synthetic chlorinated compounds frequently detected in different environmental compartments, including aquatic ecosystems [1,2,3,4]. The OCP group includes Dichlorodiphenyltrichloroethane (DDT), Aldrin, Chlordane, Dieldrin, Endrin, Heptachlor, Endosulfan and Mirex, among others, and may undergo significant bioaccumulation and biomagnification processes throughout trophic marine chains worldwide [5,6,7].The main OCP effects in fish are endocrine interference, reproductive cell degeneration and DNA damage [8,9], posing severe ecological risks and reaching humans through contaminated seafood consumption, also leading to Public Health concerns [10,11]. A shortage of assessments on the monitoring of these pollutants in coastal areas in developing countries, however, has been noted [12,13].

Although OCP were banned decades ago worldwide, these persistent organic pollutants (POPs) exhibit high environmental persistence. Their use in crops, for example leads to soil particle adsorption and transport to nearby water systems through surface run-off or groundwater flow, contaminating sediment and air, as these semi-volatiles and volatiles compounds can be carried through long distances through the wind (long-range atmospheric transport) and oceanic currents [14]. They, therefore, can accumulate in water bodies, soil, and biota for many years with slow degradation rates, linked to a range of adverse effects. Assessments concerning OCPs are, therefore, worthy of investigation, especially in developing countries such as Brazil, for example, that still allows the use of DDT for malaria and leishmaniasis control [15]. Because of this, these compounds are considered a significant threat to marine environments, especially coastal aquatic environments where exposure and levels are usually higher [16,17].

Upwelling phenomena have been reported in only certain areas worldwide [18]. This water displacement results in the emergence of deeper waters (~300 m), which are cold and rich in nutrients [19,20], increasing primary production, making upwelling areas rich in nutrients for local marine species that use the area as feeding grounds and migration routes. However, the upwelling process can also remobilize contaminants adsorbed to sediment organic matter and particulate matter to the water column and/or superficial ocean layers [21], increasing their bioavailability to marine organisms and leading to trophic chain contamination concerns. The Center-North Coast of the state of Rio de Janeiro is one such area, home to the Cabo Frio upwelling system along the continental margin towards the open sea. This area, although highly productive and one of the main fisheries landing hubs in southeastern Brazil, is located near extremely contaminated water bodies originated from several rivers that pass through industrialized areas in the state, which may contribute to high OCP contamination. Thus, a better understanding of trophic chain OCP accumulation in this area is crucial. In this context, this study aims to specifically (i) determine OCP contamination levels in various organisms belonging to different trophic levels (i.e., fish, squid, and marine mammals) in the Cabo Frio upwelling system for the first time, and (ii) ascertain the biomagnification factors of these contaminants across this trophic chain, hypothesizing that several OCP will be detected and that levels will be higher in the top component of the local trophic chain (dolphins), followed by decreasing concentrations in lower trophic levels. Our findings may offer essential insights into the ecological and public health hazards posed by OCP contamination in the Cabo Frio upwelling system and may influence future pollution control strategies.

## 2. Material and Methods

### 2.1. Chemicals

The authentic standard used for the OCP analyses was purchased from Accustandard (New Haven, CT 06513, USA) at a concentration of 1.0 ng·μL^−1^ in isooctane containing a mixture of the following pesticides: DDTs (o,p′-DDT, p,p′-DDT, o,p′-DDD, p,p′-DDD, o,p′-DDE and p,p′-DDE), Hexachlorocyclohexanes (α-, β-, γ- and δ-HCH), Chlordanes (α- and γ-chlordane, cis-chlordane, trans-chlordane, oxychlordane, heptachlor and heptachlor epoxide), Drins (aldrin, dieldrin, isodrin and endrin), Endosulfans I and II, Hexachlorobenzene (HCB), Methoxychlor and Mirex. In total, the mixture included 25 OCP. As surrogate, PCB−103 and PCB-198 (Accustandard) solutions were used, both at a concentration of 1.0 ng·μL^−1^ in order to measure the losses that occurred during the analytical process. Finally, the solution used as an internal standard (IS) for the chromatographic analysis consisted of Tetrachloro-m-xylene (TCMX, Accustandard) at 1.0 ng·μL^−1^. Purities of all standards were ≥95%. All solvents (ethanol, acetone, hexane) used in this study were HPLC grade, and chemicals were ACS grade (J.T. Baker, Phillipsburg, NJ, USA). Ultrapure water (resistivity > 18.0 MΩ) was obtained from a Milli-Q water purification system.

### 2.2. Study Area

**Figure 1:**
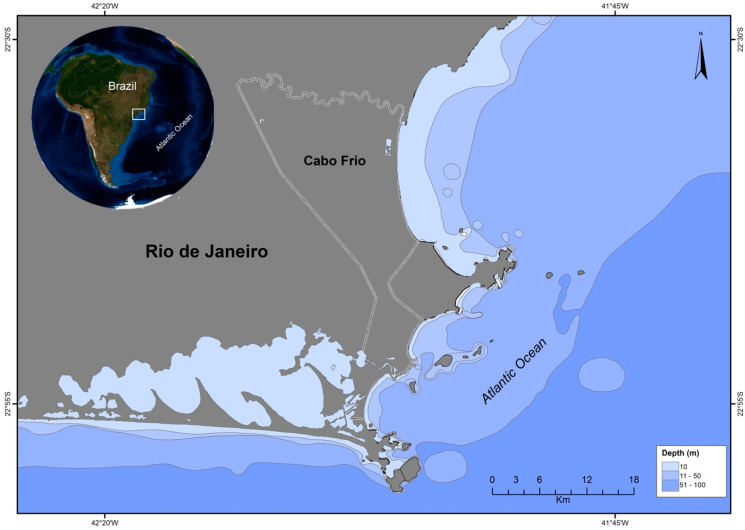
Região dos Lagos area, located in the state of Rio de Janeiro, Southeastern Brazil.

The central-northern coast of the state of Rio de Janeiro, in southeastern Brazil, comprises an important economic fisheries area, providing seafood to the entire state [22,23]. Due to increasing industrial activities, however, high chemical contamination has been noted in several local biota representatives. The Região dos Lagos area (Figure 1), for example, located 150 km from the city of Rio de Janeiro, has suffered disorderly territorial occupation and a progressive population increase since the 1970s, without the necessary infrastructure and with very low urban and environmental requirements [24].

One of the main oceanographic characteristics of the Região dos Lagos is an upwelling phenomenon, which occurs mainly during the summer due to the winds that blow in the E-NE direction, along the continental margin towards the open sea [25,26]. Thus, the Região dos Lagos is a highly productive area and the feeding grounds and migration route for many species, including marine mammals.

### 2.3. Sampling

Representative Cabo Frio upwelling system trophic chain were sampled from the Regiao dos Lagos (Figure 1), comprising nine critically endangered Franciscana dolphins (*Pontoporia blainvillei*) found stranded in the area and this species representative prey items, consisting of carnivorous cutlass fish (*Trichiurus lepturus*, *n* = 12), croaker (*Micropogonias furnieri*, *n* = 9) and squid (*Loligo plei*, *n* = 10), omnivorous mullet (*Mugil liza*, *n* = 10) and planktivorous sardines (*Sardinella brasiliensis*, *n* = 10) and chub mackerel (*Scomber japonicus*, *n* = 10) obtained from local fishers. All samples were transported on ice to the laboratory and freeze-dried for 48 h. Morphometric and sampling information are available in Tables S1 and S2. The small and isolated population of *P. blainvillei* of the northern coast of Rio de Janeiro is comprised of less than a thousand individuals and subject to high pressure of incidental catches in fishing nets and pollution [27].

### 2.4. Sample Preparation and Analysis

The freeze-dried samples were crushed and homogenized using a porcelain mortar and pestle further stored in amber bottles. The analytical method employed herein follows previously reported methods [28,29,30]. Briefly, 100 μL of the surrogate mixture (1.0 ng·μL^−1^) were added to 1 g of the sample and extracted using 20 mL of ethanol in an Ultra Turrax (model T18 basic, IKA Ltda., São Paulo, Brazil) for one min at 14,000 rpm. Sequentially, 20 mL of acetone, 20 mL of hexane and 20 mL of ultrapure water were added for extraction for one min at 14,000 rpm. After phase separation, the organic layer was transferred to a beaker using a Pasteur pipette filled with anhydrous sodium sulfate and evaporated to 1 mL using a stream of nitrogen. The extract clean-up procedure was performed by addition of 2 mL of sulfuric acid followed by vortex to breakdown residual lipids. The organic phase (extract in n-hexane) was transferred to another vial and “washed” twice with ultrapure water (purified five times with 20 mL of hexane for every two liters of water). Thus, the sample was evaporated to dryness in a water bath at a controlled temperature of 40 °C and under a light flow of high purity nitrogen, and quickly resuspended in 900 µL of hexane. Finally, 100 μL of TCMX solution (IS) at a concentration of 1.0 ng·μL^−1^ was added to the final volume of 1 mL.

The OCPs were analyzed on a gas chromatograph coupled to an electron capture detector (GC-ECD-Agilent 6890, Agilent, São Paulo, Brazil). A HP 5MS column (Agilent, São Paulo, Brazil) was used, 30 m long, 0.25 mm in diameter and 0.50 μm thick with a 5% phenylmethylsiloxane film. Hydrogen was used as the carrier gas. The temperature ramp started at 70 °C and remained in this condition for 1 min and then raised to 170 °C at 40 °C·min^−1^. Once this temperature was reached, it was again raised at a rate of 1.5 °C·min^−1^ to 240 °C and remained in this condition for 2 min. Finally, at a rate of 15 °C·min^−1^, the temperature was raised to 300 °C and remained for 5 min. The injector and detector temperatures were, respectively, 280 °C and 320 °C. Nitrogen was used as auxiliary gas (makeup), with a flow rate of 60 mL·min^−1^ and the injected volume was 2 μL in splitless mode.

### 2.5. Quality Control

For quality control, blanks were analyzed with the objective of detecting any contamination associated with the analytical process capable of making the detection and quantification of the compounds of interest unfeasible. An acceptable blank did not contain more than 3 interferents co-eluting with the analytes and their level could not be more than 3 times the limit of method detection. Blanks were injected in the beginning of the sequence and every 10 samples into the GC. Analytical curves were prepared in hexane at the following concentrations: 1, 5, 10, 20, 50, 80, 100, 150 and 200 pg·µL^−1^. The coefficient of linearity (R^2^) was equal to or greater than 0.995 for all compounds. Surrogate recovery (Rs) was calculated using the following equation:

(1) $$Rs\left(\%\right)=\left[\frac{Ca\left(s\right)\times Cs\left(IS\right)}{Ca\left(IS\right)\times Cs\left(s\right)}\right]\times 100$$

where Ca and Cs represents the average concentration and the spiked concentration, respectively. The indices (s) and (IS) stands for surrogate and internal standards, respectively. Surrogate recoveries (PCB—103 and −198) ranged from 71 to 110%. The method limits of detection (MDL) were calculated by the following equation MDL = 3 × S/m, where S is the standard deviation of the blank and m is the slope of the calibration curve. The MDL ranged from 0.19 to 0.47 ng·g^−1^ wet weight (ww). Similarly, the method quantification limits (MQL) were estimated as MQL = 10 × S/m, ranging from 0.87 (o,p′-DDE) to 2.12 (oxychlordane). MDL and MQL for all analyzed OCP are shown in Table S3.

### 2.6. Statistical Analyses

The Shapiro-Wilk and W test were performed to assess the data distribution. Due to a non-normal distribution, differences between groups were tested using the Kruskal-Wallis test, while correlations between OCP and morphometric variables were assessed by the Spearman correlation. Correlation associations were classified as very weak when 0.00 < rho < 0.19; weak when 0.20 < rho < 0.39; moderate when 0.40 < rho < 0.69; strong when 0.70 < rho < 0.89; and very strong when 0.90 < rho < 1.00 [31]. A significance level of 95% (*p* < 0.05) was adopted for all tests. The biomagnification factor was determined using the formula determined in previous studies [32,33] based on OCP mean concentrations measured in livers of predator and prey species as BMF = [contaminant] predator/[contaminant] prey.

## 3. Results and Discussion

### 3.1. OCP in Franciscana Dolphins

**Figure 2:**
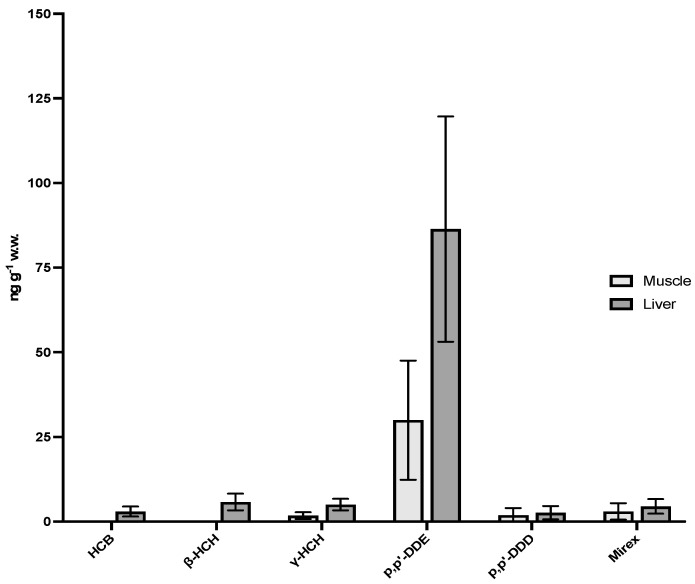
OCP concentrations in Franciscana dolphin liver and muscle samples. Data are expressed as mean ± standard deviation (ng·g^−1^ wet weight).

Among all analyzed pesticide, only p,p′-DDE, p,p′-DDD, HCB, β- and γ-HCHs and Mirex were detected above the MQL in Franciscana dolphins. DDT metabolites (p,p′-DDE and p,p′-DDD) were the predominant compounds, with the highest concentrations detected in liver (means of 2.64 and 86.4 ng·g^−1^ ww). Overall, hepatic concentrations were higher than in muscle for all detected compounds (means of 1.88 and 30.0 ng·g^−1^ ww). Organochlorine pesticide concentrations in Franciscana dolphin liver and muscle are depicted in Table S4 and Figure 2.

The presence of OCP in Franciscana dolphins in the study region is probably due to their proximity to highly urbanized and industrialized areas [24,34], receiving significant amounts of inputs from the Paraíba do Sul River, which crosses the two most industrialized states in the country, São Paulo and Rio de Janeiro, and is surrounded by thousands of industries [30,35,36]. Environmental DDT degradation takes place through chemical interactions, mainly through photoreactions or biotransformation by microorganisms present in the soil [12]. The main biotransformation DDT products are DDD and DDE congeners, which are usually more environmentally persistent than the DDT precursors [37,38]. DDTs are absorbed by exposed biota organisms, due to their highly lipophilic nature, and undergo bioaccumulation and biomagnification processes, which are mostly detected in higher trophic levels, i.e., predatory fish and marine mammals [39]. DDT was widely used in Brazil for agricultural purposes in the 1970s and early 1980s [40].

The ratio between cetacean muscle and liver p,p′-DDE and ΣDDTs concentrations is applied to evaluate whether DDT contamination is recent or not, where values above 0.60 indicate old DDT sources and below 0.60 suggest recent contamination [41]. The ratio calculated for *P. blainvillei* ranged between 0.64 and 0.94, indicating non-recent DDT sources. However, according to Leonel [42], is it not possible to verify if the transformation of p,p′-DDT into p,p′-DDE took place in the environment or in cetaceans or their prey, as high p, p′-DDE/ΣDDTs values may be also the result of p,p′-DDE-rich diet [43]. Nevertheless, the high p,p′-DDE/ΣDDTs values calculated herein indicate that the analyzed system is stabilized, with no new DDTs inputs [44]. This is in line with the restrictions and bans in the use, commercialization, and distribution of these compounds by the Brazilian government in 1985 [45]. Currently, DDT can only be used against leishmaniasis and malaria vectors and in agricultural emergency situations [12].

Following DDTs, the most abundant organochlorine pesticide was Mirex, at mean muscle and liver concentrations of 3.02 ng·g^−1^ ww and 4.51 ng·g^−1^ ww, respectively. Comparatively, Mirex levels were slightly higher than HCHs compounds in muscle, probably due to their high persistence and stability conferred by twelve chlorine atoms [46], low mobility, high hydrophobicity and no metabolization by most organisms, therefore undergoing trophic bioaccumulation and biomagnification processes [11,47].

Among HCHs, only γ-HCH (lindane) was found above the MQL in both *P. blainvillei* tissues, whereas β-HCH was detected only in liver. The β-HCH isomer was detected at mean concentrations of 5.80 ng·g^−1^ ww in liver, while the γ-HCH isomer was present at a means of 1.77 ng·g^−1^ ww and 5.04 ng·g^−1^ ww in muscle and liver, respectively. Lower HCH levels may be associated with higher elimination rates and lower persistence when compared with other pesticides [48,49]. HCH isomers are normally present in the environment in the form of gases in the atmosphere or dissolved in water and a small fraction of this compound is adsorbed on particulate matter [50]. In Brazil, lindane was mainly used as a pesticide in coffee, soybean, and cotton plantations, as well as in the control of the Chagas disease vector [51]. This chemical is still permitted as a wood conservation agent [52].

HCB was found only in dolphin livers, at a mean of 2.97 ng·g^−1^ ww. Despite its high chemical stability, HCB is relatively volatile, thus undergoing long-range transport and reaching mainly polar and/or subpolar regions [53]. Higher concentrations are commonly found in these regions compared to tropical and temperate regions [54,55]. Due to the bans on its use in the 1980s and its physicochemical characteristics, low HCB concentrations are expected in Brazil, even though it can still be obtained as a by-product of the manufacture of chlorinated solvents and new pesticides [56].

A significant strong correlation was noted only between p,p′-DDD and Mirex, indicating the same probable source (rho = 0.80). This may be due to the fact that Mirex and DDT (which is degraded into p,p′-DDD and p,p′-DDE) are still routinely used in Brazil, the former as a formicide and the latter, although banned in the country by the Ministry of Agriculture in 1995, as a pesticide in crops and for vector control measures throughout the country [57].

Dolphin exposure to OCP takes place mainly through the dietary route and influenced by feeding habits. The *Pontoporia blainvillei* diet is based on the ingestion of crustaceans, cephalopods, and small fish, usually not over 10 cm in length and not occupying high trophic levels [58,59]. The relatively low OCP concentrations detected in the *P. blainvillei* is probably due to the fact that most of the analyzed dolphins were juveniles.

However, most studies on this endangered species analyzed OCPs in blubber samples, with a lack of studies noted for liver and muscle assessments. It is not uncommon for OCP assessments to focus only on blubber, as these contaminants tend to accumulate in lipid-rich tissues [60,61]. However, other tissues must be considered when studying pollutant exposure in marine animals, and liver and muscle tissues can provide information on more recent exposure to pollutants and aid researchers in better understanding how these compounds are metabolized and distributed throughout the body.

A single study was found using liver and muscle tissues in individuals of Franciscana dolphins, sampled along the coast of São Paulo, Brazil, carried out by [62]. These authors reported the following OCP range concentrations: DDT: 1.02–9.44 ng·g^−1^ ww in liver and 0.50–6.93 ng·g^−1^ ww in muscle; Mirex: <0.147–0.433 ng·g^−1^ ww in liver and <0.059–0.083 ng·g^−1^ ww in muscle; HCB: <0.360–0.630 ng·g^−1^ ww in liver and <0.144–0.147 ng·g^−1^ ww in muscle; and HCH: <0.218–3.01 ng·g^−1^ ww in liver and <0.087 ng·g^−1^ ww in muscle. The DDT, Mirex, HCH, and HCB concentrations reported by those authors were overall lower than those detected herein. This may indicate higher OCPs contamination throughout the coast of the state of Rio de Janeiro compared to the state of São Paulo.

### 3.2. OCP in Fish and Squid

The OCP concentrations in squid and fish exhibited very similar profiles between species, with only γ-HCH and p,p′-DDE above the MQL in all samples. The only exception was noted for swordfish livers, which also presented HCB, p,p′-DDD and Mirex above the MQL (Table 1).

Similarly to the sampled dolphins, the most abundant compound in all samples was p,p′-DDE, which is much more persistent and resistant to environmental degradation than its precursor, p,p′-DDT [38]. HCB, β-HCH, some DDT derivatives and Mirex were detected above the MDL, but below MQL and therefore could not be quantified.

The highest OCP concentrations (p,p′-DDE) were detected in *T. lepturus* liver samples, probably due to its voracious predatory and cannibalistic habits [63], occupying the highest trophic level among the analyzed species. However, the highest p,p′-DDE levels in muscle were not detected in swordfish, but instead in croaker (*M. furnieri*), at a means of 34.48 ng g^−1^ ww. This species is also carnivorous, albeit based on polychaetes, bivalves and fish [64,65], that is, smaller prey than swordfish. Similarly, croaker’s livers also showed γ-HCH concentrations higher than those detected in swordfish. Similar findings have been reported by Quinete et al. [30], where higher POPs values were detected in croaker when compared to swordfish in a region close to the mouth of the Paraíba do Sul River.

As mentioned previously, the sampling area of the present study is characterized as a discharge area of extremely contaminated water bodies that pass through industrialized Rio de Janeiro, making this a priority area for contaminant monitoring. The presence of OCP in the squid and fish samples analyzed herein probably originate from the proximity of their habitat to highly urbanized and industrialized areas (OCP bioconcentration from surrounding polluted waters) and/or their diet (bioaccumulation due to the ingestion of smaller fish or invertebrates). It is known that both adult *Trichurus lepturus* and Franciscana dolphins exploit coastal waters to obtain their food resources, with a slight overlap in their feeding habits [58], which may explain the high levels detected in *T. lepturus* liver.

Brazil is, in fact, one of the world’s largest pesticide importers [66], and food, water and environment pesticide contaminations have become a serious issue in the country due to extensive applications, mainly in vast soybean, corn and cotton plantations [67,68]. According to WHO and FAO, an extraneous maximum residue limit (EMRL) of 1.0 μg kg^−1^ is established for DDT and its metabolites in food. The DDE levels found herein were higher than this limit, which seems to indicate critical coastal environment pesticide contamination [69], implying in potential deleterious health effects for the studied species and the ecosystem as a whole. Furthermore, most of the sampled fish species are highly consumed in the study area, representing a major exposure risk to local residents, and also sold to other Rio de Janeiro state regions implying potential human health risks. Potential OCP exposure health effects in humans include, but are not limited to, different types of cancer, neurological disorders, neurodegenerative diseases, diabetes, and decreased fertility, due to significant endocrine disruptor properties [70].

The general OCP concentrations detected in *T. lepturus*, *M. furnieri*, *M. liza* and *S. brasiliensis* were higher than those reported by da Silva et al. [71] in fish from Guanabara Bay, Rio de Janeiro, and similar to those reported by Caldas et al. [72] for fish from Paranoá Lake, in Brasília, Central-West Brazil. Specifically concerning DDTs and HCHs, levels were very similar than those reported by Liebezeit et al. [73] and by Miranda et al. [13] in the state of Paraná, south Brazil. Interestingly, Caldas et al. [72] established a comparison with studies carried out in the 1970s in the same location and verified a decline in the concentrations of DDTs, HCHs, Drins and Chlordanes in Paranoá Lake. Finally, squid showed levels of γ-HCH and p,p′-DDE similar to those found by Ueno et al. [74] in Japan, Santhi et al. [75] off the coast of Malaysia and Storelli et al. [76] on the Italian coast. However, although some assessments have been conducted on OCP contamination of some of the same fish species analyzed herein in the state of Rio de Janeiro [77,78], this is the first study of its kind in the Cabo Frio upwelling region, and also the first to assess distinct trophic levels.

Concerning Spearman correlations, significant correlations were detected between p,p′-DDE and weight in *M. furnieri* muscle (rho = −0.74), and *T. lepturus* muscle (rho = 0.66), indicating higher accumulation with age, between p,p′-DDD and p,p′-DDE in *T. lepturus* liver (rho = 0.76), indicating the probable source and, thus, metabolization.

### 3.3. Biomagnificaton Factors (BMF)

Di Beneditto and Ramos [79] determined that crustaceans are present in only 25% of Franciscana dolphin diets (*n* = 85), suggesting lesser dietary importance, while cephalopods were recorded in 66% of analyzed Franciscana dolphin stomachs. Three Loliginidae species were identified: *Loligo sanpaulensis*, *Loligo plei* and *Lolliguncula brevis*. Teleost fish were the most representative items, recorded in 95% of the analyzed stomachs. Twenty species from six families were identified, including *Micropogonias furnieri*, *Trichiurus lepturus*, *Sardinella brasiliensis*. Cutlass fish, in turn, prey on many crustaceans, cephalopods, and teleosts, including the Mugil genus, also assessed herein [63], while *Scomber japonicus* and *Sardinella brasiliensis* are both planktivorous.

Based on the liver OCP concentrations of two representative prey fish species (*Trichiurus lepturus* and *Micropogonias furnieri*), the Franciscana dolphin calculated BMFs, which quantify biomagnification from successive preys to their predators along a trophic chain or food web, were 0.18 for γ-HCH, 0.68 for mirex, 1.78 for p,p′-DDD and 1.37 for p,p′-DDE. The cutlass fish BMF based on mullet consumption was estimated as 0.58 for γ-HCH and 1.34 for p,p′-DDE.

The BMFs of 1.78 for p,p′-DDD and 1.37 for p,p′-DDE indicate biomagnification of these compounds throughout the analyzed food web to dolphins, while p,p′-DDE was noted as biomagnifying up to cutlass fish. Lower BMF for HCH compared to DDT degradation products have been previous noted when employing blubber [80]. The results shown here suggest that OCP biomagnification in liver samples is not as pronounced as in more fatty tissues, which have shown BMFs from 3–213 times in Indo-Pacific humpback dolphins (*Sousa chinensis*) and their prey fishes from the Pearl River Estuary, China [80]. Other possible explanations for the low BMF are biodilution effects, hepatic clearance or OCP transfer to fetus during pregnancy and lactation (for female individuals, *n* = 5). For fish, similar trophic transfer factor was observed for DDT (1.04) in Pacific oyster (*Crassostrea gigas*) to goby (*Acanthogobius hasta*) and for HCHs (0.68) in shore crab (*Hemigrapsus penicillatus*) to goby [7]. Additional studies are needed for a better assessment of the biomagnification/trophic transfer of organochlorinated pesticides in top predator species, which would provide critical information on the potential risks to marine mammal and fish health.

## 4. Conclusions

Several OCPs were detected in Franciscana dolphin and fish samples from the Cabo Frio upwelling system trophic chain. DDE, a DDT degradation product, was the OCP with highest concentrations found in both liver and muscle samples of *P. blainvillei*, with higher concentrations in liver. The same trend was observed in all species of fish, except for *M. furnieri*. Calculated BMFs in Franciscana dolphins indicate that OCPs in the study region biomagnify throughout the food web from prey (*T. lepturus* and *M. furnieri*) to these top predators. We highlight the lack of studies investigating OCP exposure in Franciscana dolphins, especially in muscle and liver samples. Therefore, it is important that future research consider multiple tissues when studying OCP exposure in marine mammals to obtain a more comprehensive scenario of the effects of these compounds on these individuals and what that may represent at the population level. Biomagnification processes are in place for both DDT metabolites in dolphins and for p,p′-DDE in cutlass fish, even though non-recent DDT sources were noted, indicating concerns. Therefore, continued OCP monitoring in this region is also warranted to better understand the distribution and fate of these compounds over time, with the goal to establish strategies for the conservation of local dolphin species and assess human health risks in local coastal region populations.

## Figures and Tables

**Table 1:** OCP concentrations in squid and fish muscle and liver samples. Data are expressed as mean ± standard deviation (ng·g^−1^ wet weight).

Species	*n*	Tissue	HCB	γ-HCH	p,p′-DDE	p,p′-DDD	Mirex
*Loligo plei*	10	Muscle	<MQL	3.75 ± 2.01	22.6 ± 11.5	<MQL	<MQL
*Scomber japonicus*	10	Muscle	<MQL	1.37 ± 0.71	19.1 ± 5.7	<MQL	<MQL
*Sardinella brasiliensis*	10	Muscle	<MQL	2.04 ± 1.08	4.47 ± 2.67	<MQL	<MQL
*Mugil liza*	10	Muscle	<MQL	4.33 ± 2.28	6.69 ± 4.32	<MQL	<MQL
		Liver	<MQL	6.64 ± 1.70	8.97 ± 3.36	<MQL	<MQL
*Micropogonias furnieri*	9	Muscle	<MQL	4.12 ± 4.56	34.5 ± 15.6	<MQL	<MQL
		Liver	<MQL	18.2 ± 12.7	26.8 ± 7.1	<MQL	<MQL
*Trichiurus lepturus*	12	Muscle	<MQL	6.19 ± 8.51	26.3 ± 9.7	<MQL	<MQL
		Liver	1.70 ± 0.93	10.6 ± 5.2	36.1 ± 20.6	1.48 ± 0.79	6.61 ± 3.91

Legend: MQL = Method quantification limit.

## Data Availability

Data is available upon reasonable request.

## Supplementary Materials

The following supporting information can be downloaded at: https://www.mdpi.com/article/10.3390/ijerph20146343/s1,: Table S1: Morphometric and collection information for *Pontoporia blainvillei* individuals; Table S2: Morphometric and collection information for fish species and squid; Table S3: Method detection limits (MDLs) and method quantification limits (MQLs) in ng g^−1^ wet weight for all analyzed organochlorinated pesticides (OCPs); Table S4: Average concentrations and range in muscle and liver samples of *Pontoporia blainvillei* (*n* = 9).
