# Supplementary material for: Trophic Chain Organochlorine Pesticide Contamination in a Highly Productive Upwelling Area in Southeastern Brazil
Source: Int J Environ Res Public Health. 2023 Jul 11;20(14):6343. doi: 10.3390/ijerph20146343 (PMC10379595; doi:10.3390/ijerph20146343)
Supplement: Supplementary file 1 [file ijerph-20-06343-s001.zip › ijerph-2355976-supplementary.pdf]

TROPHIC CHAIN ORGANOCHLORINE CONTAMINATION IN A HIGHLY PRODUCTIVE UPWELLING AREA IN SOUTHEASTERN BRAZIL

[1]Ricardo Lavandier, [2]Jennifer Arêas, [3]Leila Lemos, [4]Jailson de Moura, [5]Satie Taniguchi, [5]Rosalinda Montone, [6,7]Natalia Quinete, [8]Rachel Ann Hauser-Davis, [9]Salvatore Siciliano, [1]Isabel Moreira

[1]Departamento de Química, Pontifícia Universidade Católica do Rio de Janeiro (PUC-Rio), Rua Marquês de São Vicente, 225, Gávea – Rio de Janeiro, RJ 22453-900, Brazil
[2] PIBIC/Fiocruz, Av. Brasil, 4.365, Manguinhos, Rio de Janeiro, RJ 21040-900, Brazil
[3]Institute of Environment, Florida International University, Miami, FL, USA
[4] Systems Ecology, Leibniz Center for Tropical Marine Ecology (ZMT), Fahrenheitstrasse 6, 28359 Bremen, Germany
[5]Instituto Oceanográfico, Universidade de São Paulo (USP), Praça do Oceanográfico 191, Butantã, São Paulo, SP 05508-900, Brazil
[6] Institute of Environment, Florida International University, Miami, FL, USA
[7] Department of Chemistry and Biochemistry, Florida International University, 11200 SW 8th Street, Miami, FL 33199, USA
[8] Laboratório de Avaliação e Promoção da Saúde Ambiental, Instituto Oswaldo Cruz, Fundação Oswaldo Cruz, Av. Brasil, Rio de Janeiro, Manguinhos 4365, Brazil
[9] Departamento de Ciências Biológicas, Escola Nacional de Saúde Pública/Fiocruz Av. Brasil, 4.365, Manguinhos, Rio de Janeiro, RJ 21040-900, Brazil

Supplementary Information:

Table S1. Morphometric and collection information for Pontoporia blainvillei individuals

Table S2. Morphometric and collection information for fish species and squid

Table S3. Method detection limits (MDLs) and method quantification limits (MQLs) in ng g^{-1} wet weight for all analyzed organochlorinated pesticides (OCPs)

Table S4. Average concentrations and range in muscle and liver samples of *Pontoporia blainvillei* (n=9)

Table S1. Morphometric and collection information for Pontoporia blainvillei individuals

Sample ID	Length (cm)	Sex	Collection Location	Collection Status	Date
PB01	144	Female	Sao Joao da Barra	accidental capture	7/30/2011
PB02	125	Female	Sao Joao da Barra	accidental capture	1/16/2012
PB03	144	Female	Sao Joao da Barra	stranded	1/24/2012
PB04	136	Male	Quissama	stranded	2/9/2012
PB05	122	Male	Quissama	stranded	7/9/2012
PB06	113	Female	Quissama	stranded	8/28/2012
PB07	115	Male	Quissama	accidental capture	8/31/2012
PB08	102	Female	Quissama	accidental capture	9/23/2012
PB09	107	Male	Quissama	accidental capture	9/25/2012

Table S2. Morphometric and collection information for fish species and squid

Species	Sample ID	Length (cm)	Sex	Collection Location	Date
Trichiurus Lepturus	PE01	117	female	Cabo Frio	8/14/2012
	PE02	148	female	Cabo Frio	8/14/2012
	PE03	121	female	Cabo Frio	8/14/2012
	PE04	118	male	Cabo Frio	8/14/2012
	PE05	133	female	Cabo Frio	8/14/2012
	PE06	119	female	Cabo Frio	8/14/2012
	PE07	115	female	Cabo Frio	8/14/2012
	PE08	108	female	Cabo Frio	8/14/2012
	PE09	136	female	Cabo Frio	8/14/2012
	PE10	140	female	Cabo Frio	8/14/2012
	PE11	105	female	Cabo Frio	8/14/2012
	PE12	119	male	Cabo Frio	8/14/2012
Micropogonias furnieri	C01	48	female	Cabo Frio	8/15/2012
	C02	53	female	Cabo Frio	8/15/2012
	C03	50	female	Cabo Frio	8/15/2012
	C04	47	male	Cabo Frio	8/15/2012
	C05	50	female	Cabo Frio	8/15/2012
	C06	57	female	Cabo Frio	8/15/2012
	C07	49	female	Cabo Frio	8/15/2012
	C08	53	female	Cabo Frio	8/15/2012
	C09	47	female	Cabo Frio	8/15/2012
Mugil liza	T01	56	female	Cabo Frio	8/15/2012
	T02	54	female	Cabo Frio	8/15/2012

	T03	55	male	Cabo Frio	8/15/2012
	T04	60	female	Cabo Frio	8/15/2012
	T05	58	female	Cabo Frio	8/15/2012
	T06	50	male	Cabo Frio	8/15/2012
	T07	54	female	Cabo Frio	8/15/2012
	T08	57	female	Cabo Frio	8/15/2012
	T09	57	female	Cabo Frio	8/15/2012
	T10	54	male	Cabo Frio	8/15/2012
Sardinella brasiliensis	S01	24	N/A	Praia Seca	9/24/2013
	S02	24	N/A	Praia Seca	9/24/2013
	S03	25	N/A	Praia Seca	9/24/2013
	S04	26	N/A	Praia Seca	9/24/2013
	S05	25	N/A	Praia Seca	9/24/2013
	S06	24	N/A	Praia Seca	9/24/2013
	S07	25	N/A	Praia Seca	9/24/2013
	S08	24	N/A	Praia Seca	9/24/2013
	S09	24	N/A	Praia Seca	9/24/2013
	S10	25	N/A	Praia Seca	9/24/2013
Scomber japonicus	CAV01	31	N/A	Praia Seca	9/25/2013
	CAV02	32	N/A	Praia Seca	9/25/2013
	CAV03	30	N/A	Praia Seca	9/25/2013
	CAV04	28	N/A	Praia Seca	9/25/2013
	CAV05	27	N/A	Praia Seca	9/25/2013
	CAV06	30	N/A	Praia Seca	9/25/2013
	CAV07	31	N/A	Praia Seca	9/25/2013
	CAV08	30	N/A	Praia Seca	9/25/2013
	CAV09	27	N/A	Praia Seca	9/25/2013
	CAV10	29	N/A	Praia Seca	9/25/2013
Logigo plei	L01	36	N/A	Cabo Frio	8/15/2012
	L02	33	N/A	Cabo Frio	8/15/2012
	L03	42	N/A	Cabo Frio	8/15/2012
	L04	37	N/A	Cabo Frio	8/15/2012
	L05	41	N/A	Cabo Frio	8/15/2012
	L06	37	N/A	Cabo Frio	8/15/2012
	L07	34	N/A	Cabo Frio	8/15/2012
	L08	42	N/A	Cabo Frio	8/15/2012
	L09	39	N/A	Cabo Frio	8/15/2012
	L10	38	N/A	Cabo Frio	8/15/2012

Table S3. Method detection limits (MDLs) and method quantification limits (MQLs) in ng g^{-1} wet weight for all analyzed organochlorinated pesticides (OCPs)

OCPs	MDL	MQL
α-HCH	0.310	1.40
HCB	0.300	1.36
β-HCH	0.430	1.91
γ-HCH	0.360	1.63
δ-HCH	0.410	1.82
Heptachlor	0.22	0.97
Aldrin	0.22	0.99
Isodrin	0.330	1.48
Heptachlor Epoxide A	0.420	1.88
Oxychlordane	0.470	2.12
Heptachlor Epoxide B	0.410	1.86
γ-Chlordane	0.340	1.54
o,p'-DDE	0.19	0.87
Endosulfan I	0.270	1.21
α-Chlordane	0.45	2.0
Dieldrin	0.230	1.03
p,p'-DDE	0.27	1.20
o,p'-DDD	0.20	0.91
Endrin	0.280	1.23
Endosulfan II	0.420	1.88
p,p'-DDD	0.290	1.31
o,p'-DDT	0.310	1.39
p,p'-DDT	0.280	1.24
Methoxychlor	0.320	1.44
Mirex	0.410	1.84

Table 4. Average concentrations and range in muscle and liver samples of Pontoporia blainvillei (n=9)

Compounds	Muscle		Liver	
	**Average ± SD**	**Range**	**Average ± SD**	**Range**
HCB	<MQL	<MQL	2.97 ± 1.48	2.11 - 6.01
β-HCH	<MQL	<MQL	5.80 ± 2.46	2.84 - 10.09
γ-HCH	1.77 ± 1.01	1.64 -3.00	5.04 ± 1.71	3.48 - 9.14
p,p'-DDE	29.97 ± 17.58	13.69- 40.19	86.38 ± 33.26	42.56 - 120.58
p,p'-DDD	1.88 ± 2.08	<MQL- 5.72	2.64 ± 1.95	<MQL- 5.91
Mirex	3.02 ± 2.43	1.93 - 7.29	4.51 ± 2.15	2.12 - 8.67

SD: standard deviation
